# A prospective cohort study identifying risk factors for shoulder injuries in adolescent elite handball players: the Karolinska Handball Study (KHAST) study protocol

**DOI:** 10.1186/s12891-017-1852-2

**Published:** 2017-11-22

**Authors:** Martin Asker, Markus Waldén, Henrik Källberg, Lena W. Holm, Eva Skillgate

**Affiliations:** 1 Musculoskeletal & Sports Injury Epidemiology Center, Institute of Environmental Medicine, Karolinska Institutet, Stockholm, Sweden; 2 Naprapathögskolan - Scandinavian College of Naprapathic Manual Medicine, Stockholm, Sweden; 3 Department of Medical and Health Sciences, Division of Community Medicine, Linköping University, Linköping, Sweden; 4 Department of Orthopaedics, Hässleholm-Kristianstad-Ystad Hospitals, Hässleholm, Sweden; 5 Department of Monitoring and Evaluation, Public Health Agency of Sweden, Solna, Sweden; 6 Institute of Health Policy, Management and Evaluation, University of Toronto, Toronto, ON M5T 3M6 Canada

**Keywords:** Athletic injuries, Elite, Handball, Overhead sport, Overuse, Risk factor, Shoulder injury

## Abstract

**Background:**

Handball is a physical contact sport that includes frequent overhead throwing, and this combination leads to a high rate of shoulder injuries. Several factors have been associated with shoulder injuries in overhead athletes, but strong scientific evidence is lacking for most suggested risk factors. We therefore designed the Karolinska Handball Study (KHAST) with the aim to identify risk factors for shoulder injuries in adolescent male and female elite handball players studying at handball-profiled secondary schools in Sweden. Secondary objectives are to investigate whether shoulder function changes during the competition season and whether the physical profile of the players changes during their time in secondary school.

**Methods:**

Players aged 15 to 19 years were included during the pre-season period of the 2014–2015 and the 2015–2016 seasons. At inclusion, players signed informed consent and filled in a questionnaire regarding playing position, playing level, previous handball experience, history of shoulder problems and athletic identity. Players also completed a detailed test battery at baseline evaluating the shoulder, neck and trunk. Players were then prospectively monitored weekly during the 2014–2015 and/or 2015–2016 competitive seasons regarding injuries and training/match workload. Results from the annual routine physical tests in the secondary school curriculum including bench press, deep squat, hand grip strength, clean lifts, squat jumps, counter movement jumps, <30 m sprints, chins, dips and Cooper’s test will be collected until the end of the competitive season 2017–2018. The primary outcome is the incidence of shoulder injuries and shoulder problems. The secondary outcome is the prevalence of shoulder injuries and shoulder problems.

**Discussion:**

Shoulder problems are frequent among handball players and a reduction of these injuries is therefore warranted. However, in order to introduce appropriate preventive measures, a detailed understanding of the underlying risk factors is needed. Our study has a high potential to identify important risk factors for shoulder injuries in adolescent elite handball players owing to a large study sample, a high response rate, data collection during consecutive seasons, and recording of potential confounding factors.

**Electronic supplementary material:**

The online version of this article (10.1186/s12891-017-1852-2) contains supplementary material, which is available to authorized users.

## Background

Handball is an Olympic team sport that includes both physical contact and frequent overhead throwing, which stress the shoulder girdle [1–3]. A high frequency of shoulder injuries has therefore been reported previously in both male and female youth and senior players [4–8]. Several factors, such as decreased shoulder mobility, shoulder weakness and scapula dyskinesia, have been associated with shoulder injuries in studies of overhead athletes [6, 9–13]. Strong scientific evidence is, however, lacking for the importance of most of the suggested risk factors in the literature. The importance of the findings of common clinical screening methods is also questioned [14, 15].

In order to identify possible risk factors, large high-quality prospective cohort studies are required with a careful registration of potential confounding factors at baseline and thorough follow-up [14, 16]. Most of the recent studies on risk factors for handball injuries have included senior players and there is, to our knowledge, no prospective study meeting these aforementioned criteria that has investigated potential risk factors for shoulder injuries in adolescent elite handball players. Since recent studies has shown that shoulder injuries in adolescent players is as common as in senior players, [4, 12] and the prevalence of shoulder problems in senior players is high and often persistent or recurrent, [5, 6, 8] a greater understanding of injury characteristics and injury prevention in youth players could also have a positive impact on the prevalence of injury in senior players as well. Additionally, no study has investigated if factors such as shoulder strength, shoulder range of motion (ROM), scapula dyskinesia, trunk mobility and shoulder joint position sense (JPS) change in adolescent elite players during a handball season.

We therefore designed the Karolinska Handball Study (KHAST) with the aim to identify risk factors for shoulder injuries in adolescent male and female elite handball players studying at handball-profiled secondary schools in Sweden. Secondary objectives are to investigate whether shoulder function changes during the competition season and whether the physical profile of the players changes during their time in secondary school.

## Methods/design

### Study design

This is a prospective cohort study designed in accordance with the Strengthening the Reporting of Observational Studies in Epidemiology (STROBE) guidelines [17]. The study does not evaluate any intervention and a trial registration was therefore not needed.

### Study setting

Male and female handball players studying at secondary schools in Sweden with a handball-profiled programme certified by the Swedish Handball Federation (SHF) were eligible for participation in this study. In total, there were 38 such schools with approximately 1100 students at the time of the project start in 2014. For the purpose of this study, the following inclusion criteria were applied: (i) the schools needed to have a capacity for at least 35 handball-profiled students, (ii) the schools needed to have an even enrolment of male and female players, and (iii) the schools needed to be classified by the SHF as being at the highest elite level. The criteria of having the capacity for at least 35 players was convenient and set to ensure that a satisfactory number of study participants would be reached during the limited pre-season time window. Further, we wanted the schools to have both female and male players enrolled, since we wanted to investigate and compare risk factors for both genders. Ten schools met these criteria and all these eligible schools accepted to participate in this study.

### Study participants

Out of a total of 552 eligible players aged 15 to 19 years, 471 (54% females) were included. Two hundred and seventy-four players were included and followed in 2014–2015 and 197 players were included and followed in 2015–2016. Out of the 274 who were included and followed in 2014–2015, 151 were also tested and followed again in 2015–2016, which results in a total of 622 players-seasons (out of 703 eligible player seasons) in the prospective follow-up. A summary of the measurements during the different phases of the study is presented in Table 1.

**Table 1:** Summary of the measurements during the different phases of the KHAST

Phase	Measurements	Equipment/tools
Baseline (September 2014 and 2015)	Gender. Match and training exposure, injury history, handball level, playing position, athletic identity. Shoulder ROM, stability, strength and JPS. Neck control. Trunk mobility. Scapular dyskinesis.	Modified OSTRC overuse injury questionnaire Fahlström’s questionnaire AIMS questionnaire KHAST test protocol
Follow-up September 2014 to May 2015 and September 2015 to May 2016	Weekly reports on injuries and training and game load.	Modified OSTRC overuse injury questionnaire
Follow-up on subgroup September 2014 to May 2015 and September 2015 to May 2016	Weekly reports on injuries and training and game load. Bi-monthly measurements; Shoulder ROM, stability, strength and JPS. Neck control. Trunk mobility. Scapular dyskinesis.	Modified OSTRC overuse injury questionnaire KHAST test protocol
Follow-up once every semester 2014–2018	Physical profile including; bench press, deep squats, Cooper’s test, 10 and 20-m sprint, squat jumps, CMJ, hand grip strength, clean lifts, chins, dips, weight and height.	Handgrip dynamometer, timing gates, measuring tape, scale and gym equipment

*AIMS* Athletic Identity Measurement Score, *CMJ* Counter movement jumps, *JPS* Joint position sense, *KHAST* The Karolinska Handball Study, *OSTRC* Oslo Sports Trauma Research Center, *ROM* range of motion

### Baseline questionnaire

The pre-season KHAST baseline questionnaire was based on Fahlström’s questionnaire, [18, 19] and the validated and translated Swedish version of the Oslo Sports Trauma Research Center (OSTRC) overuse injury questionnaire [20, 21]. For the baseline questionnaire, we modified the OSTRC overuse injury questionnaire in that the players were asked about shoulder problems during the preceding season and the past two months instead of the past week. The baseline questionnaire was completed by the players at baseline in September 2014 or 2015 and focused on playing position, playing level, previous handball experience (match and training history), and history of shoulder injuries. Additionally, it also included the Athletic Identity Measurement Scale (AIMS) aiming to measure athletic self-identity [22]. Finally, the KHAST baseline questionnaire included questions about participation in other sports and specific training of the shoulder during the preceding season.

### Baseline test protocol

Players were also measured at baseline according to the KHAST pre-season test protocol described in detail in the electronic supplementary data. The test protocol included different measurements of strength, mobility, stability and joint position sense (JPS) of the shoulder, scapular kinesis, neck control and trunk rotational mobility [see Additional file 1]. Prior to the tests, the players completed a standard 8-min warm-up programme for the shoulders with rubber-tubes, including rotation, abduction, flexion and extension movements with graded loading.

The tests were conducted at each school’s sport facilities, either in a gym or at a handball court. There were six test stations with 1–2 test leaders at each station, depending on the test to be performed (see additional file 1). The same test leader or pair of the leaders performed each test throughout the study except for shoulder range of motion (ROM) and JPS measure, where two of the test leaders were replaced during the second season due to moving (2015–2016). In total 8 test leaders were involved and all were well experienced with the test procedure. Before data collection started, all the test leaders performed several test runs of the complete test protocol. Additionally, in order to investigate whether the shoulder function changes during the season from baseline, a convenience sample of three of the ten schools (147 players) were repeatedly measured with the KHAST test protocol every second month during one or two of the competitive seasons studied.

#### Shoulder strength

Isometric external and internal rotation shoulder strength and eccentric external rotation shoulder strength was measured with a hand-held dynamometer (MicroFet2, Hoggan Health Industries Inc. West Jordan, UT, USA) in a seated position with the arm in the frontal plane in 90° [23, 24]. Isometric abduction was measured with the player standing with the arm in 30° abduction in the scapular plane [6]. Two tests were performed in each direction and the highest value will be used for analysis.

#### Shoulder mobility

Passive glenohumeral rotational ROM of both the dominant and non-dominant shoulder was measured using a goniometer, with the player in a supine position with the shoulder in 90° abduction and elbow flexed to 90° [23]. One tester fixated the scapula with one hand and rotated the arm with the other hand until movement of the scapula was felt under the hand. Another tester measured the degrees of shoulder rotation using a goniometer. Three repetitions were performed in each direction and the mean value of these three measurements will be used for analyses.

#### Shoulder stability and laxity

To evaluate shoulder stability the apprehension test, load and shift test and test for sulcus sign was performed with player in a seated position. The test for sulcus sign was performed both with the shoulder in neutral position and externally rotated [25, 26]. The apprehension test was performed with the player’s arm in 90° abduction in the frontal plane and maximum rotated and was considered positive if the player experienced any discomfort in the shoulder or apprehend during the test [27]. The load and shift test was graded from 1 to 3 and all these categories will be used in the analyses. The sulcus sign was judged as present or not present [25].

#### Shoulder joint position sense

Shoulder JPS of the dominant shoulder was measured with the patient blind folded, in supine position. A digital inclinometer (Mini Digital Protractor, ODT tools, Vimmerby, Sweden) was used to measure degrees of rotation in the shoulder [23]. The player started with the arm in 90° abduction and 90° flexion in the elbow. A target angle (TA) was set at 75% of the player’s maximum passive external rotation. The tester passively rotated the arm to the TA and instructed the player to keep the arm in that position for 3 s before the arm was passively returned to the starting position. The player was then instructed to actively rotate the arm back to the TA and the tester measured the angle and registered the difference from the TA. This procedure was repeated three times and the mean error of the TA from the three attempts was recorded and will be used in the analyses [28].

#### Scapular kinesis

For the observation of scapular dyskinesia each player did two repetitions of maximum shoulder abduction and two repetitions of maximum shoulder flexion in random orders with weights, in standing position. Male players lifted 2-kg weights and female players lifted 1-kg weights. The test was video recorded and one test leader later watched all the recorded videos and judged scapular dyskinesia as present or not present [29].

#### Neck control

Neck control was evaluated using a modification of the cranio-cervical flexion test, using a stabilizer (Stabilizer Pressure Bio-Feedback, Chattanooga group Inc.) [30]. In this modified test the players was instructed to slightly push the neck against the stabilizer to increase the pressure and keep the pressure 3 × 10 s at a specific level, starting at 22 mmHg. If the players could perform this task they were instructed to increase the pressure to 24 mmHg and keep the pressure for another 3 × 10 s. This was repeated with a 2 mmHg increase until the player reached 30 mmHg. The last level that the players were able to complete a full set of 3 × 10 s contraction was used for analysis. A more detailed description of the modification of this test is presented in the additional material [see Additional file 1].

#### Trunk mobility

Trunk rotational mobility was measured in a seated position on a graded, 10 mm thick gym mat, with the legs crossed over each other [31]. For this study the mat was graded with 5-grades steps instead of 45° that has been used in a recent study [31]. The players were instructed to sit with their legs crossed and a strait posture and then slowly rotate as long as they could to each side. Three repetitions were performed in each direction and the highest values will be used for analysis.

### Physical profile

In addition to this pre-season KHAST test protocol, all the schools perform physical tests every year as part of their conventional curriculum, including bench press, deep squat, hand grip strength, clean lift, squat jump, counter movement jump, 10 and 20 m sprints, maximum amount of chins and dips and Cooper’s 12 min run test [32]. These test results will be collected annually from study start until Spring 2018, which would be at the end of the competitive season of 2017–2018.

### Injury registration and monitoring of handball exposure

All included players were followed weekly with web-based questionnaires. The injury and exposure data were collected prospectively from September 3rd 2014 to April 30th 2015 and from September 4th 2015 to April 24th 2016.

In the weekly follow-up reports, the players responded to the Swedish version of the OSTRC overuse injury questionnaire [21]. The questionnaire includes four questions concerning the consequences of problems in the shoulder, with 4–5 answer alternatives. For the purpose of this study, we added one answer option to the first question; “Could not participate/reduced participation due to another reason than shoulder problems”. If the player ticked this box he/she had to specify the reason why. The questions were preceded by a short introduction explaining that all questions should be completed, regardless of whether or not the players had experienced any problems in that area, and giving examples of the most common overuse symptoms. The survey software prohibited questionnaire submission of incomplete reports. In the weekly follow-up reports, questions regarding match and training exposure (including both training on the handball court and other training outside the court, respectively) and a question about acute injuries were also asked.

The players received an email with a link to the online weekly report each Sunday during the follow-up period. The players were encouraged to fill in the questionnaire during school hours once a week to reach a high participation rate. If no response had been received from the player, they were automatically sent a reminder email the next day and daily until Wednesday where those who still had not answered received a cellular phone reminder via a short message service (SMS) with the link to the weekly report. If a player also failed to respond to the SMS reminder, they were contacted by telephone by a research assistant. If a player reported an acute injury, regardless of anatomical site, they were contacted by telephone by an experienced clinician for further questions regarding the injury type, situation, anatomical site, time-loss due to the injury, if they have received any medical care due to the injury and in that case what diagnosis they received.

### Outcome measurements

The primary outcome is incidence of shoulder injuries and shoulder problems. Each answer in the OSTRC Overuse Injury Questionnaire corresponds to a score and for each question a score between 0 and 25 could be given, which leads to a sum score between 0 and 100 for the total questionnaire [20]. Shoulder injuries will be defined as reporting a score of 40 or more in the OSTRC Overuse Injury Questionnaire at some point during the season. Shoulder problems will be defined as any or substantial shoulder problems. If a player reports any problems e.g. reports anything but the lowest grades of the four questions in the OSTRC overuse injury questionnaire this will be categorized as any shoulder problems. For substantial shoulder problems we will use the same definition as originally described “Players who reported (shoulder) problems leading to moderate or severe reductions in training volume, or moderate or severe reductions in sports performance or complete inability to participate in sport (i.e., athletes who selected option 3, 4 or 5 in either Question 2 or Question 3)” [20].

Secondary outcomes are; 1) time-loss shoulder injury, defined as numbers of consecutive weeks with substantial shoulder problems as defined above, 2) the baseline, weekly and season prevalence of shoulder problems, 3) changes in shoulder function during the competitive season measured in the subgroup, 4) changes in physical profile from season to season.

### Sample size

No previous data on injury incidence and normative values from the test protocol exist for handball players in the studied age group. Based on previous data from senior elite handball players [5, 6], however, using a power of 80%, a significance level of 5%, a drop-out rate of 10% and an expected rate ratio of 1.5 for the primary outcome, we calculated that approximately 500 players was needed with a follow-up period of one competitive season.

### Statistics

The injury incidence will be calculated as number of injuries per 1000 playing hours. The season prevalence will be calculated by dividing the number of players reporting shoulder problems at some point with the total number of players included in the study. Similarly, the week prevalence will be calculated by dividing the number of players who report shoulder problems during the week with the number of players who responded to that weekly report. Generalized linear models will be performed to investigate any differences in prevalence of shoulder problems among gender, playing position, secondary school grade and playing level and will be presented as a prevalence ratio (PR) with corresponding 95% confidence interval (CI). Cox regression models will be performed to investigate if the factors from the baseline questionnaire and the pre-season test protocol are risk factors for shoulder injuries. Exposed players will be compared to unexposed players with consideration taken to potential confounding. Based on the Cox regression models, hazard rate ratio (HRR) will be presented with corresponding 95% CI. Further, we plan to use mixed models to investigate whether shoulder function and/or physical profile change over time (repeated measures).

### Ethics

The Regional Ethics Review Board of the Karolinska Institutet, Stockholm, Sweden, approved the study (2013/1722–31/4). All participating players, and legal guardians when appropriate, gave written informed consent when entering the study. None of the authors were involved in diagnosis or treatment of player injuries.

## Discussion

Shoulder injuries and shoulder problems are frequent and disabling in male and female handball players [5, 6, 8], and a reduction of these injuries and problems are highly warranted. The results from this study on risk factors for shoulder injuries will therefore be of importance for current athlete screening, load monitoring, injury management and future injury prevention in the sport and possibly in other overhead sports as well.

### Methodological considerations

There are a number of methodological strengths with this study compared with the current handball and overhead sports literature. First, the major strength of this study is the large sample size with 471 individual players and 622 player-seasons included. Second, the sample is also likely to be representative of the population of adolescent elite handball players in Scandinavia, which gives our study a high external validity. Third, we will collect robust data of potential confounding factors, which is essential for a future valid identification of possible risk factors for injury. Fourth, our study has almost the same distribution of girls and boys, which will enable analyses of potential gender-related differences in shoulder injury characteristics and underlying risk factors.

Some limitations of this study must also be noted. First, even though the exposure of match minutes and training hours is reported every week, the intensity of training is not measured specifically which could be of importance when analysing playing load. However, although this is of great interest, we believe that a weekly questionnaire that is too extensive, could potentially result in attrition, and thus affect the response rate negatively. Second, even though the competitive season had not started when the players were tested in September, they had trained handball for several weeks since the start of the pre-season in August. This means that some players may already had developed shoulder problems before the baseline assessments and could thus influence the incidence measures. Third, it is not possible to document any objective and specific shoulder diagnoses with our registration method and this limits a more detailed analysis of the shoulder injury characteristics. Fourth, there is a risk that the players who are measured repeatedly during the competitive season will get used to the tests, i.e. there is a potential “learning effect” and that this will influence the test results. Finally, although we measured many potential risk and confounding factors, we did not include factors such as, for example, sleeping habits and mental stress at baseline, which also could be of relevance in the studied age group.

## Additional file

Additional file 1: The KHAST test protocol. (PDF 560 kb)

## Abbreviations

AIMS: Athletic Identity Measurement Scale
JPS: Joint Position Sense
KHAST: The Karolinska Handball Study
OSTRC: Oslo Sports Trauma Research Center
ROM: Range Of Motion
SHF: Swedish Handball Federation
SMS: Short Message Service
STROBE: Strengthening the Reporting of Observational studies in Epidemiology
TA: Target Angle
