# Supplementary material for: A prospective cohort study identifying risk factors for shoulder injuries in adolescent elite handball players: the Karolinska Handball Study (KHAST) study protocol
Source: BMC Musculoskelet Disord. 2017 Nov 22;18:485. doi: 10.1186/s12891-017-1852-2 (PMC5700469; doi:10.1186/s12891-017-1852-2)

THE KAROLINSKA HANDBALL STUDY - KHAST

Test protocol

Shoulder strength

MUSIC
Musculoskeletal & Sports Injury Epidemiology Center

- Isometric internal rotation (IR) and external rotation (ER) strength was measured using a handheld dynamometer (HHD), (MicroFet2, Hoggan health Industries Inc., West Jordan, UT, USA) that was placed 2 cm proximal to the styloid process of the ulna with the players in a seated position with the arm in frontal plane in 90° abduction and 90° flexion in the elbow.

- The tester fixated the shoulder and arm of the players with one hand and forearm and held the HHD with the other hand. The players were then instructed to push against the HHD during 5 seconds, submaximal in the beginning building it up to a maximum isometric force.

- The eccentric strength in external rotation was measured with the players in the same position as for external isometric strength using the HHD. The tester then pushed the arm into internal rotation while the players were instructed to resist the motion. A metronome was used to help the tester to keep the right phase, 30°/s.

- Abduction strength was measured in a standing position with the arm in scapular plane with 30° abduction. Tape and a large goniometer were used to align the player's arm in the right position.

- Two tests were performed in each direction with ten seconds rest between each test and the maximal values were used for analysis. The order of test positions was randomized.

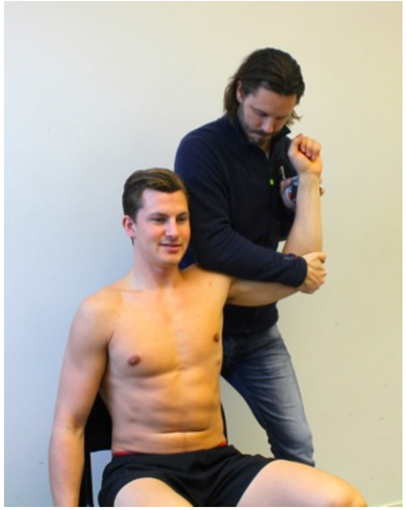

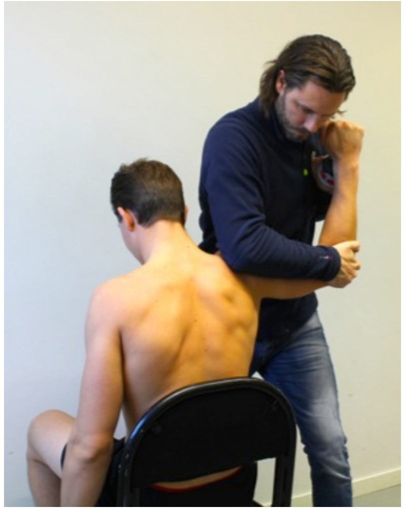

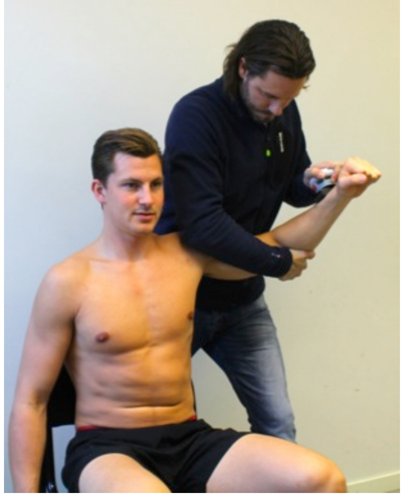

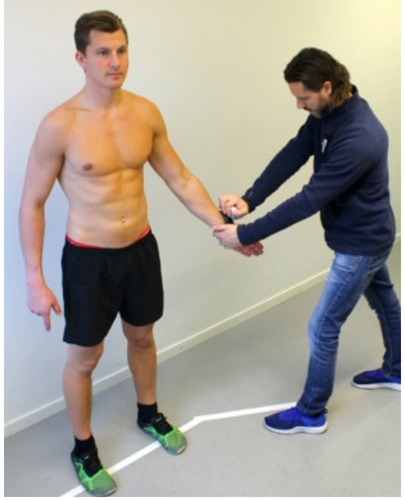

Trunk mobility

MUSIC
Musculoskeletal & Sports Injury Epidemiology Center

- The players were seated on a stiff carpet with their legs crossed holding a stick on the shoulders with crossed arms. If the players had trouble sitting with their legs crossed they were allowed to sit on an 8 cm stiff mat. The carpet was graded, with five grades steps, from zero to one hundred and eighty degrees.

- The players were instructed to sit with a strait posture and then slowly rotate as long as they could to each side.

- Three repetitions were performed to one side and then three repetitions to the other side and the tester registered the degrees of rotation by using a long steel liner. The highest value in each direction was registered as rotation to dominant side or non-dominant side, where the player's throwing shoulder was defined as dominant

- The order of the direction of rotation was randomized.

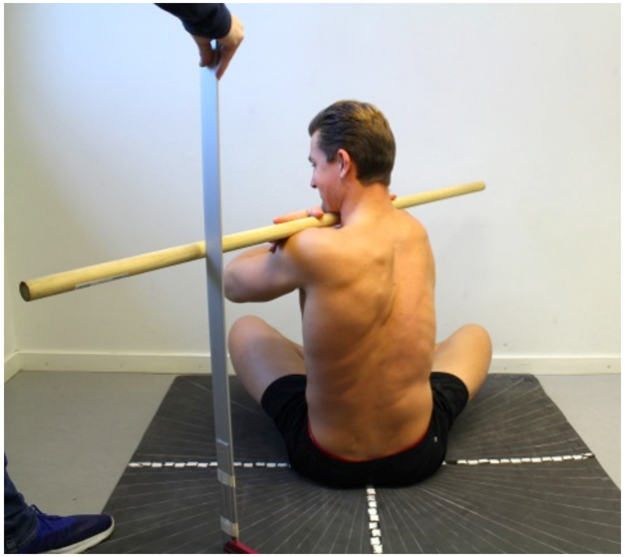

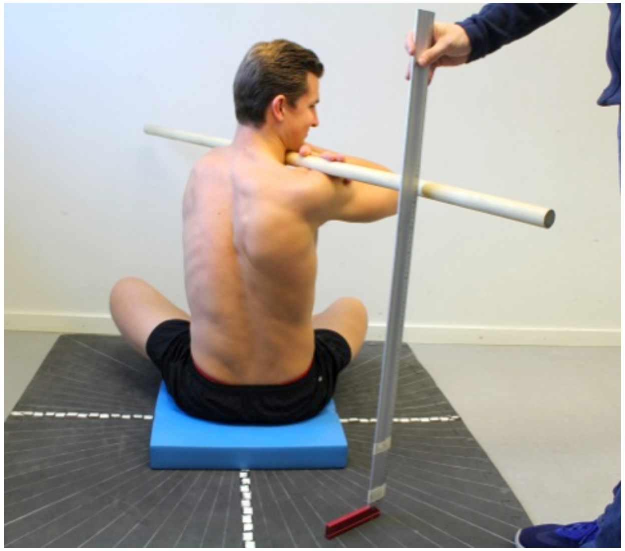

Cranio-cervical flexion control

MUSIC
Musculoskeletal & Sports Injury Epidemiology Center

- A modification of the cranio-cervical flexion test was used, grading the performance from 22-30 mmHg or not able to perform the test.
- The players laid in a supine position on a treatment table with their hips flexed and the feet on the table and hands on the stomach. A stabilizer (Stabilizer Pressure Bio-Feedback, Chattanooga group Inc.) was put under the player's neck and the tester held the display so both the players and the tester could read it. The test was divided into two parts, a pre-test screening and the actual test.
- For the screening test the players were instructed to slightly push the neck against the stabilizer up to a pressure of 22 mmHg and hold that pressure for five seconds and then relax.
- If the players were able to do this, they were instructed to increase the pressure to 24mmHg and hold that pressure for five seconds. This procedure was repeated for 26 mmHg, 28 mmHg and 30 mmHg adding up in total five seconds contraction at each level.
- If no pain was present during this pre-test screening and they were able to complete all steps the actual test was carried out.

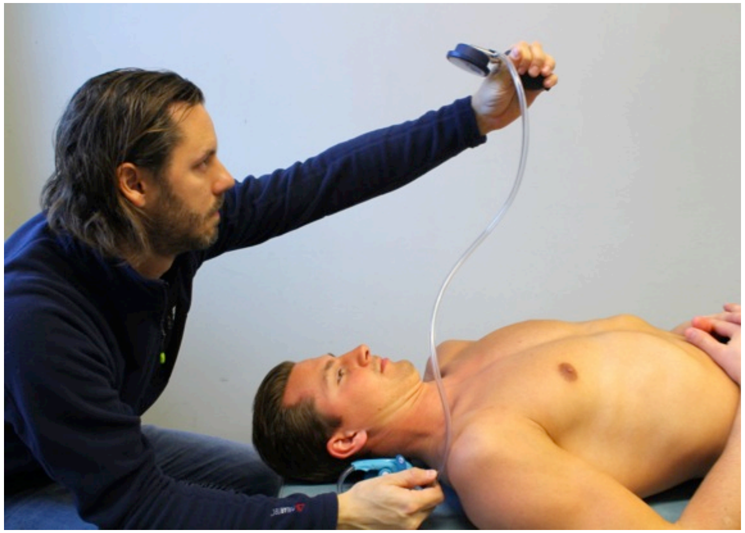

Cranio-cervical flexion control (continuing)

- During the actual test the same test position and instructions were used as during the pre-test screening but now the players had to keep the same pressure for 3x10 seconds before moving on to the next level, with a ten second rest between each set.

- The level that the players were able to complete a 3x10 seconds contraction was recorded and used for analysis. For instance if a player managed to maintain a pressure of 26 mmHg for 3x10 seconds but only 2x10 seconds on 28 mmHg, 26 mmHg was the score that was recorded.

- With this modification the players could get either; incomplete (not passing the pre-test screening) or 22 mmHg, 24 mmHg, 26 mmHg, 28 mmHg or 30 mmHg as a test score.

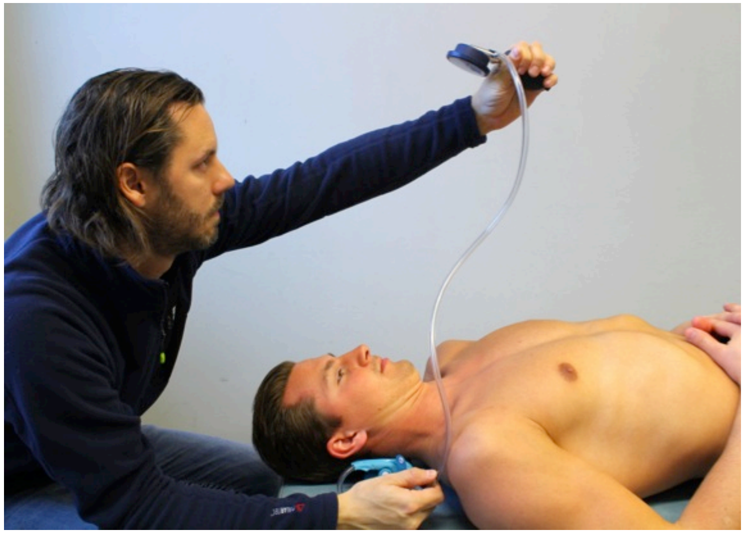

Glenohumeral range of motion (ROM)

MUSIC
Musculoskeletal & Sports Injury Epidemiology Center

- Glenohumeral range of motion was measured in random order with the players in supine position with the shoulder in 90° abduction and 90° flexion in the elbow.
- One of the testers firmly fixed the scapula with one hand, with the thumb on the coracoid process and the fingers on scapular spine. With the other hand the tester internally respectively externally rotated the shoulder until the tester felt the movement of the scapula under his/her hand.
- The other tester aligned one of the goniometer's arms horizontally and the other arm along the player's forearm using the styloid process of the ulna as a reference point and then read the goniometer. The test was repeated twice for each direction and the mean value was registered.
- Right after the internal and external ROM was measured the players were instructed to do a submaximal isometric contraction in external and internal rotation for 3x5 seconds against the tester resistance and then the measurement for internal and external rotation ROM was repeated as described above.

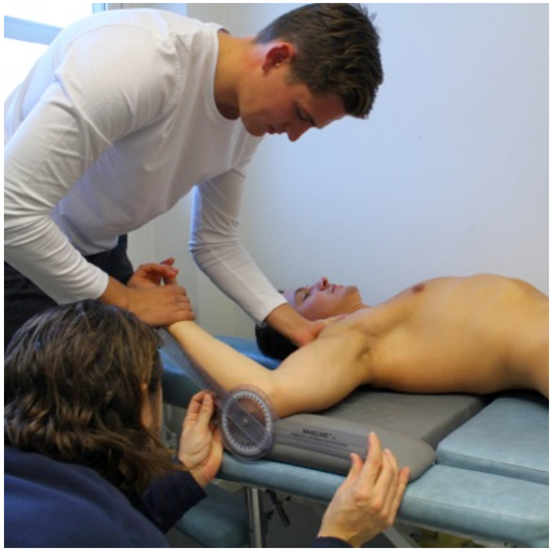

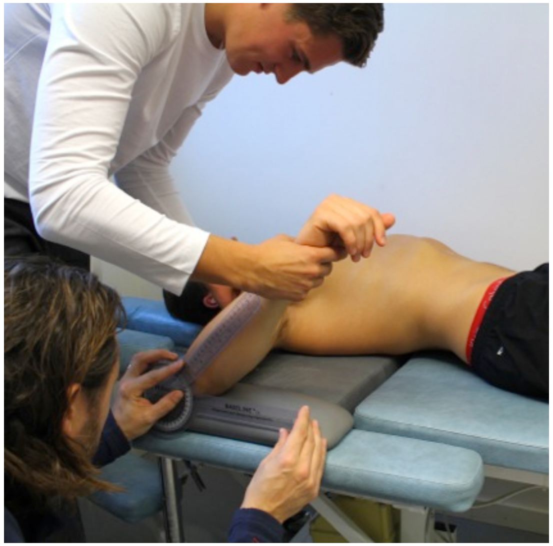

Scapular kinesis

MUSIC
Musculoskeletal & Sports Injury Epidemiology Center

- A headlight was used to standardize the light setting for each test environment. The test was video recorded from a posterior view from a standardised distance of 3 meters.

- For the observation of scapular dyskinesia each player did two repetitions of maximum shoulder abduction and two repetitions of maximum shoulder flexion in random orders with weights, in standing position. The female players used a one-kilogram dumbbell and male players used a two-kilogram dumbbell.

- The players were instructed to lift their arms as high up as possible with their thumbs pointing upward. One tester later observed the videos and judged scapula dyskinesia as “Yes or No”.

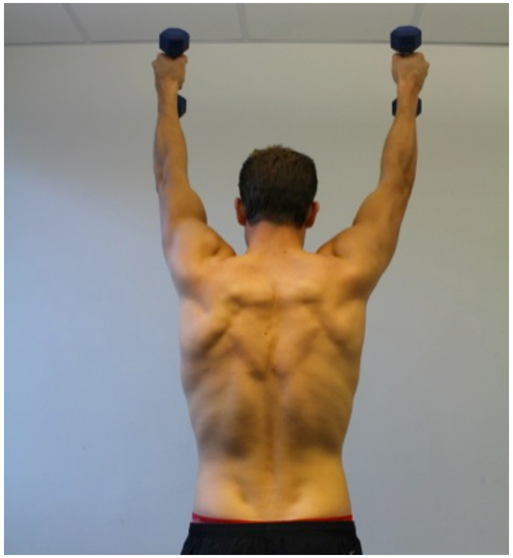

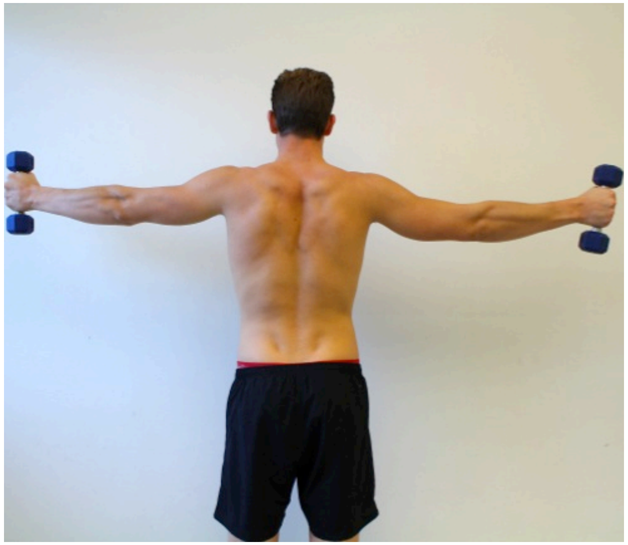

Shoulder joint reposition sense (JPS)

MUSIC
Musculoskeletal & Sports Injury Epidemiology Center

- The JPS in the dominant shoulder was measured with the players in supine position.
- The target angle (TA) for the JPS test was set to 75% of maximum external rotation and tested with the arm in 90° abduction and 90° flexion in the elbow. A digital inclinometer (Mini Digital Protractor, ODT tools, Vimmerby, Sweden) was used to measure degrees of rotation.
- From the starting position the tester passively rotated the players arm to the TA and the players were instructed to hold that position without support for 3 seconds before the tester passively return the arm to the starting position.
- The players were then instructed to rotate the arm back to the TA and the tester measured the angle and the difference from the TA was registered (JPS error).
- The measurement was repeated three times and a mean value JPS error from the TA of the three recordings was used for analysis.

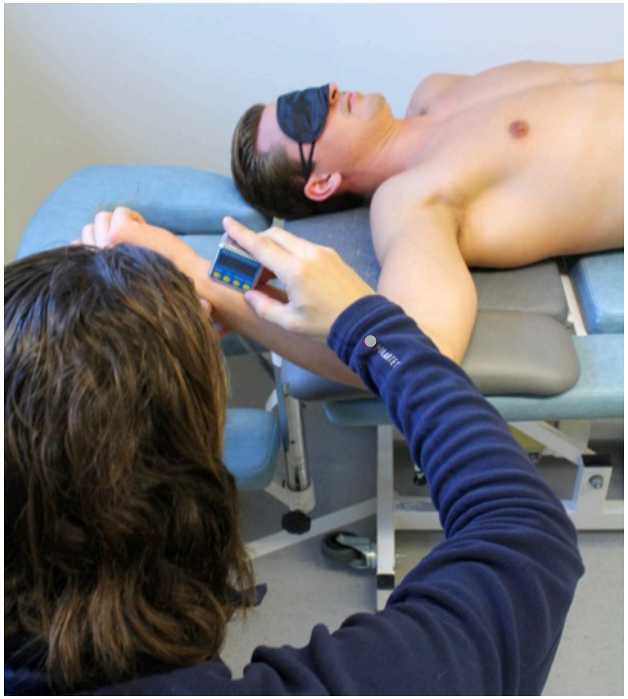

Supplement: Additional file 1: — The KHAST test protocol. (PDF 560 kb) [file 12891_2017_1852_MOESM1_ESM.pdf]
